# Haptoglobin genotype predicts development of coronary artery calcification in a prospective cohort of patients with type 1 diabetes

**DOI:** 10.1186/1475-2840-10-99

**Published:** 2011-11-20

**Authors:** Melissa Simpson, Janet K Snell-Bergeon, Gregory L Kinney, Orit Lache, Rachel Miller-Lotan, Yefim Anbinder, Marian J Rewers, Andrew P Levy

**Affiliations:** 1 Barbara Davis Center for Childhood Diabetes, Aurora CO, USA; 2 Technion Faculty of Medicine, Technion Israel Institute of Technology, Haifa, Israel

**Keywords:** Cardiovascular disease, type 1 diabetes mellitus, coronary artery calcium, hyperglycemia, genetics, Haptoglobin

## Abstract

**Background:**

Coronary artery disease has been linked with genotypes for haptoglobin (Hp) which modulates extracorpuscular hemoglobin. We hypothesized that the Hp genotype would predict progression of coronary artery calcification (CAC), a marker of subclinical atherosclerosis.

**Methods:**

CAC was measured three times in six years among 436 subjects with type 1 diabetes and 526 control subjects participating in the Coronary Artery Calcification in Type 1 Diabetes (CACTI) study. Hp typing was performed on plasma samples by polyacrylamide gel electrophoresis.

**Results:**

The Hp 2-2 genotype predicted development of significant CAC only in subjects with diabetes who were free of CAC at baseline (OR: 1.95, 95% CI: 1.07-3.56, p = 0.03), compared to those without the Hp 2-2 genotype, controlling for age, sex, blood pressure and HDL-cholesterol. Hp 2 appeared to have an allele-dose effect on development of CAC. Hp genotype did not predict CAC progression in individuals without diabetes.

**Conclusions:**

Hp genotype may aid prediction of accelerated coronary atherosclerosis in subjects with type 1 diabetes.

## Background

Coronary artery disease (CAD) is the leading cause of death in patients with type 1 diabetes and CAD mortality is 2-4 higher among type 1 diabetes patients than in subjects without diabetes [1,2]. While hyperglycemia and conventional cardiovascular risk factors contribute to this increased risk, they do not account for all of the excess risk. Therefore additional markers are needed to predict which individuals with type 1 diabetes are at greatest risk for developing CAD [3]. Coronary artery calcium (CAC) is a powerful marker of the coronary artery plaque burden [4]. Both the presence and progression of CAC have been shown to predict CAD events [5]and mortality [6].

Haptoglobin (Hp) is a protein whose primary function is to modulate the fate and toxicity of extracorpuscular hemoglobin [7]. The Hp protein is polymorphic with two classes of alleles, designated 1 and 2. In most populations of European ancestry, the prevalence of the Hp 1-1 genotype is < 20%; Hp 2-1 and Hp 2-2 have approximately equal frequencies [8]. The protein products of the Hp 1 and Hp 2 alleles are structurally and functionally distinct. The Hp 1 protein mediates more rapid clearance of free hemoglobin and provides superior protection against hemoglobin-driven oxidation compared to the Hp 2 protein [7].

Studies in patients with type 2 diabetes have reported a 2-5 fold increased risk of myocardial infarction, stroke or CAD death in patients with the Hp 2-2 genotype, compared to those without the Hp 2-2 genotype [9-13]. In patients with type 1 diabetes, the Hp 2-2 genotype conferred an approximately 2-fold increased risk of CAD, compared to Hp 1-1, with an intermediate risk found in Hp 2-1 individuals [9].

The association between the Hp genotype and CAC has not been previously examined. The purpose of this study was to investigate the Hp genotype as a predictor of CAC progression in adults with and without type 1 diabetes. Based on previous prospective studies of CAD, we hypothesized that the Hp 2-2 genotype would predict CAC progression in patients with type 1 diabetes, but not in those without diabetes.

## Methods

### Study participants

The Coronary Artery Calcification in Type 1 Diabetes (CACTI) study enrolled 1,416 individuals between 19 and 56 years of age, with no known history of CAD: 652 participants with type 1 diabetes and 764 control participants without diabetes. Twenty five people were excluded because they had a coronary event. Of those remaining, 172 individuals with type 1 diabetes and 216 controls have not completed the 6 year follow-up examination. Additional subjects were excluded because of missing plasma sample (n = 28) or inability to determine the Hp genotype (n = 13), leaving 436 subjects with diabetes and 526 controls in the analyses. The baseline CAD risk factors for the CACTI participants included in the analyses did not differ from those who were excluded, except for younger age of excluded controls. Participants with type 1 diabetes had long-standing disease (mean duration ± SD: 23 years ± 9 years) at baseline, were insulin dependent within 1 year of diagnosis, and were diagnosed prior to age 30 years or had positive antibodies or a clinical course consistent with type 1 diabetes. All study participants provided informed consent and the study protocol was approved by the Colorado Multiple Institutional Review Board.

### Coronary Artery Calcium Measurement

At each examination, an ultrafast Imatron C-150XLP EBCT scanner (Imatron, San Francisco, CA) was used to obtain two sets of high resolution, noncontrast, contiguous 3-mm tomographic images acquired at 100-ms exposure. Scanning started from near the lower margin of bifurcation of the main pulmonary artery with the subject breathholding for ~35-45 s and proceeded caudally. Calcified coronary artery areas were identified as those with a minimum density of 130 Hounsfeld units (HU) and a minimum area of three pixels (1.03 mm2). A calcium score for each region was calculated by multiplying the area by the density score (1 for 130-199, 2 for 200-299, 3 for 300-399, and 4 for > 399 HU). A total CAC score in Agatston units (AU) was calculated by adding up scores for all slices separately for left main, left anterior descending, circumflex, and right coronary arteries [14]. The scanner was recalibrated every day with a phantom. Effective radiation dose for an EBCT sequence was 1.0 mSV for men and 1.3 mSV for women [15].

The presence of any CAC at baseline was defined as a CAC score > 0 on either of the two scans. CAC progression was defined as a change in square root CAC volume ≥ 2.5, as this difference was determined to be < 1% likely to be due to measurement error, based on the two scans that were completed within 5 minutes of one another [16]. The same CAC measurement protocol was followed at the baseline and 6 year follow up visit.

### Haptoglobin typing

Hp typing was performed on stored plasma samples by polyacrylamide gel electrophoresis as previously described [17]. Briefly, 10 ul of Hb enriched plasma was subjected to electrophoresis in a non-denaturing gel and the gel was subsequently immersed in solution containing a congener of benzidine with a precipitate forming in the gel corresponding to the location of Hb-Hp complexes. The Hp type of the sample was determined by the banding pattern of the Hp-Hb complexes with each of the three Hp types having a characteristic banding fingerprint. Previous work has established a 1:1 correspondence between this method and a PCR based method for Hp genotyping [17]. An unambiguous Hp type was obtained on 98.7% of all samples. For the purposes of quality and control and validation, we simultaneously measured the Hp type using an ELISA based assay [7,18] with a greater than 97% agreement in the Hp type assigned by the two methods.

### Statistical analysis

We used two analytic approaches to examine the association between CAC and Hp polymorphism: linear regression and logistic regression. For both analyses, we considered the following baseline CAD risk factors as covariates: sex, age, diastolic blood pressure (mmHg), systolic blood pressure (mmHg), body mass index (Kg/m^2^) (BMI), low-density lipoproteins (LDL), high-density lipoproteins (HDL), triglycerides, hemoglobin A1c (%), self-reported history of ever smoking, and baseline square root transform of the CAC volume among those with CAC present at the baseline examination. We used backwards selection to build the most parsimonious model; this selection process entails removing the covariate that has the largest p-value given the other covariates in the model until all covariates included have a p value < 0.05. Any covariate whose p-value was not < 0.05 in the multivariate model was not included in the final adjusted model. Assessment of baseline CAD risk factors have been previously described [15]. The outcome in linear regression analyses was the change in square root transformed CAC volume over the 6 year follow up period. For logistic regression analyses, CAC progression was defined as a change in the square root transformed CAC volume ≥ 2.5 over the 6 year period of follow-up, which corresponds to a change in CAC volume from 0 to ≥ 6.25 among participants initially free from CAC. This cut off was chosen because it is the point at which the change in CAC is significant enough that it can not be attributed to interscan variability [16]. All analyses were performed using SAS for Windows version 9.2 (SAS Institute Inc., Cary, NC, USA).

## Results

Table 1 describes the demographic characteristics of the study population stratified by diabetes status and CAC progression over a mean time between the baseline and the follow up visit of 6.5 years (± 0.6, minimum: 4, maximum: 9). In the univariate comparison, the frequency of the Hp 2-2 polymorphism did not differ by CAC progression (p = 0.13 in participants with type 1 diabetes and p = 0.17 in those without diabetes).

**Table 1:** Baseline characteristics in study subjects with 6 year progression data and Hp genotype data by diabetes status

Characteristic	Study subjects with type 1 diabetes			Control subjects without type 1 diabetes		
	
	6 Year CAC Progression ≥ 2.5	6 Year CAC Progression < 2.5		6 Year CAC Progression ≥ 2.5	6 Year CAC Progression < 2.5	
	
	n = 178	n = 258		n = 150	n = 376	
CAC present at baseline visit	113(63)	37(14)	*	86(57)	51(14)	*
Haptoglobin genotype 2-2	76(43)	89(35)		46(31)	139(37)	
Mean 6 year change in CAC volume	7.4 ± 4.4	0.4 ± 0.8	*	5.8 ± 3.1	0.3 ± 0.7	*
Female	78(44)	160(62)	*	41(27)	223(59)	*
Mean age (years)	40.1 ± (8.1)	34.0 ± 8.2)	*	45.2 ± 7.3)	39.0 ± 8.5	*
Mean diastolic blood pressure (mmHg)	78.0 ± 9.0	76.3 ± 8.4	*	83.2 ± 8.9	77.2 ± 7.6	*
Mean systolic blood pressure (mmHg)	120.2 ± 13.6	113.9 ± 12.1	*	120.4 ± 12.7	111.6 ± 11.1	*
Mean body mass index (kg/m^2^)	26.7 ± 4.3	25.6 ± 4.0	*	28.2 ± 4.8	25.3 ± 4.4	*
Mean LDL cholesterol (mmol/l)	2.7 ± 0.73	2.5 ± 0.7	*	3.1 ± 0.8	3.0 ± 0.8	*
Mean HDL cholesterol (mmol/l)	1.4 ± 0.4	1.5 ± 0.4	*	1.2 ± 0.3	1.4 ± 0.4	*
Mean triglycerides (mmol/l)	1.1 ± 0.01	1.0 ± 0.5	*	1.8 ± 1.2	1.3 ± 0.7	*
Mean HbA1c (%)	8.0 ± 1.2	7.8 ± 1.2		5.6 ± 0.4	5.4 ± 0.4	*
Ever smoker	48(27)	39(15)	*	27(18)	86(23)	

* p-value less than 0.05 comparing CAC progression in individuals with type 1 diabetes and control subjects separately

Data are presented as mean ± sd or as n (%)

### Linear regression of the change in square root transform CAC volume

The Hp 2-2 polymorphism predicted change in CAC volume only in subjects with type 1 diabetes who were free of CAC at the baseline visit (Table 2, bold type). The findings were similar with adjustment for age and sex only (p = 0.03) and when additionally adjusting for systolic blood pressure and HDL-cholesterol (p = 0.05). Hp polymorphism was not associated with change in CAC volume in subjects with type 1 diabetes with CAC present at baseline or in subjects without diabetes, regardless of CAC extent at baseline.

**Table 2:** Adjusted β estimates from linear regression analyzing the association between Hp genotype and square root transformed CAC volume, stratified by the presence of CAC at baseline

		Adjusting for age and sex only		Including covariates from backwards selection model*	
Characteristic		β estimate	*p*-value	β estimate	*p*-value
Hp genotype * diabetes interaction*presence of CAC at baseline interaction		N/A	0.001	N/A	0.001
CAC not present at the baseline visit	Hp 2-2 vs. 2-1/1-1 in patients with diabetes	**0.82**	**0.03**	**0.74**	**0.05**
	Hp 2-2 vs. 2-1/1-1 in controls without diabetes	0.67	0.2	0.83	0.11
CAC present at the baseline visit	Hp 2-2 vs. 2-1/1-1 in patients with diabetes	-0.34	0.3	-0.31	0.33
	Hp 2-2 vs. 2-1/1-1 in controls without diabetes	-2.02	0.52	0.24	0.7
Age		0.11	< 0.0001	0.1	< 0.0001
Female vs. male		-1.17	< 0.0001	0.22	0.001
Systolic blood pressure		N/A		0.01	< 0.0001
HDL cholesterol		N/A		0.01	0.003

* Adjusting for age, sex, systolic blood pressure and HDL-cholesterol

### Logistic regression of 6 year CAC progression

Similar to the above findings, the Hp 2-2 polymorphism predicted development of CAC (a change in CAC volume from 0 to ≥ 6.25 or in square root transformed CAC volume from 0 to ≥ 2.5) only in subjects with type 1 diabetes free of CAC at the baseline examination when adjusting for age and sex (OR: 2.03, 95% CI: 1.13-3.65, p = 0.02), and when additionally adjusting for systolic blood pressure and HDL cholesterol (OR: 1.95, 95% CI: 1.07-3.56, p = 0.03) compared to those without the Hp 2-2 polymorphism (Table 3). Hp polymorphism did not predict CAC progression in individuals without diabetes.

**Table 3:** Adjusted odds ratios for the association between CAC progression and Hp genotype, stratified by presence of CAC at baseline

		Adjusting for age and sex only			Including covariates from backwards selection model*		
Characteristic		OR	95% CI	*p*-value	OR	95% CI	*p*-value
Hp genotype * diabetes interaction*presence of CAC at baseline interaction		N/A		0.03	N/A		0.09
CAC not present at the baseline visit	Hp 2-2 vs. 2-1/1-1 in patients with diabetes	**2.03**	**1.13-3.65**	**0.02**	**1.95**	**1.07-3.56**	**0.03**
	Hp 2-2 vs. 2-1/1-1 in controls without diabetes	0.83	0.37-1.84	0.65	0.69	0.38-1.27	0.94
CAC present at the baseline visit	Hp 2-2 vs. 2-1/1-1 in patients with diabetes	0.68	0.37-1.24	0.21	0.80	0.33-1.93	0.22
	Hp 2-2 vs. 2-1/1-1 in controls without diabetes	1.33	0.94-3.28	0.07	1.17	0.49-2.81	0.29
Age		1.08	1.05-1.10	< 0.0001	1.08	1.05-1.10	0.009
Female vs. male		0.43	0.31-0.60	< 0.0001	0.65	0.45-0.94	< 0.0001
Systolic blood pressure		N/A			1.03	1.02-1.05	< 0.0001
HDL cholesterol		N/A			0.97	0.96-0.99	0.0001
Square root CAC volume at baseline		N/A			1.25	1.13-1.38	< 0.0001

* Adjusting for age, sex, systolic blood pressure and HDL-cholesterol

**Figure 1:**
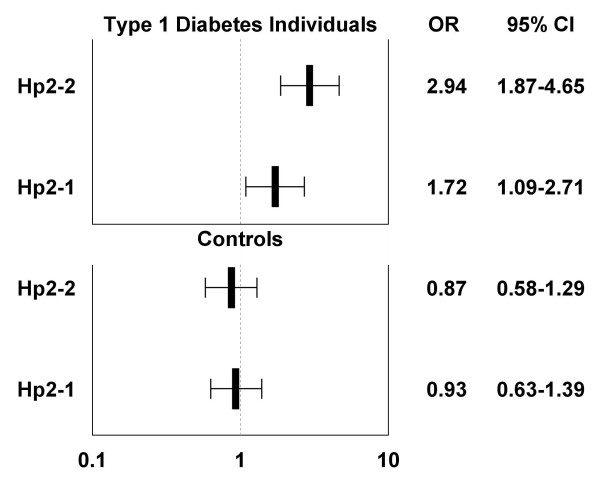
**Plot of age and sex-adjusted odds ratios (OR) for incident CAC by the number of Hp 2 alleles, stratified by type 1 diabetes**.

Among patients with diabetes free of CAC at baseline, Hp 2 appeared to have an allele-dose effect on development of CAC: Hp 2-1 OR: 1.72 (1.09-2.71) and Hp 2-2 OR: 2.94 (1.87-4.65), compared to Hp 1-1 and adjusting for age and sex (Figure 1).

## Discussion

The major finding of this study is that the Hp 2-2 polymorphism, and to a lesser extent the 2-1 polymorphism, predicts development of coronary artery calcification in people with type 1 diabetes over a period of 6 years. The findings were internally consistent, using two definitions of CAC progression in linear and logistic regression analyses. Our finding that Hp polymorphisms predict new CAC only in patients with diabetes is consistent with previous reports from studies using clinical CAD endpoints [9,10,12,13].

Consistent with our findings, the Pittsburgh Epidemiology of Diabetes Complications Study of patients with type 1 diabetes, has shown an allele-dose effect of the Hp 2 allele on the risk of incident CAD [9]. Thus, among individuals with type 1 diabetes, the Hp 2 allele plays an important role in subclinical coronary atherosclerosis and progression to clinical events.

The apparent lack of effect of Hp genotype on progression of CAC in patients already CAC positive is puzzling and requires further evaluation. While we adjusted for baseline extent of CAC in the models among patients already CAC positive, it is possible that residual confounding by the level of baseline CAC remains. In addition, incident CAC and CAC progression may reflect different biological processes and prognoses. The development of CAC is a process which increases risk for future acute coronary events, [5] and CAC progression is a powerful predictor of mortality even among persons who already have CAC present; [6] However, calcification of a plaque itself does not promote plaque rupture [19].

No other cohorts with data about incident CAC in individuals with type 1 diabetes exist in which to validate our findings. However, the results of this study validate those from studies of clinical endpoints by using the subclinical outcome of CAC, thereby demonstrating that the Hp genotype is a robust biomarker for atherosclerosis in individuals with type 1 diabetes.

The different effect of Hp 1 and Hp 2 proteins on cardiovascular risk in patients with diabetes potentially derives from several mechanisms. First, Hp regulates the fate and toxicity of extracorpuscular hemoglobin [7]. Upon binding to hemoglobin, the Hp 1 protein is superior to the Hp 2 protein in protecting against oxidation mediated by Hb derived iron [20] and HDL dysfunction, [21] particularly in the setting of diabetes. In addition, recent evidence suggests a more diverse physiologic role for Hp. Delanghe *et al. *summarized the evidence that Hp polymorphisms play a role in the regulation of both T- and B- cells, particularly with respect to the immune response to atherosclerosis and Hb driven lipid oxidation [22]. Other recent publications presented data suggesting that Hp (both genotype and circulating concentrations) has a role in remodeling the myocardium and, therefore, prognosis after myocardial infarction (MI) [23,24]. To help elucidate these mechanisms, future observational research may want to study the association between Hp concentration and CAC development as well as the interaction between the immunologic profile of people with type 1 diabetes and Hp.

## Conclusions

This study adds to the literature concerning the increased risk for CAD among patients with type 1 diabetes who have the Hp 2 allele and extends that research by studying progression of subclinical atherosclerosis rather than clinical CAD events. In so doing, this study has identified a sub-group of people towards whom primary prevention efforts may be directed. For example, the ICARE study found that vitamin E is useful in prevention of clinical cardiovascular events among individuals with type 2 diabetes and the Hp 2-2 polymorphism [25,26]. Therefore, it may be useful to initiate a similar clinical trial targeted at Hp 2-2 individuals with type 1 diabetes and no CAC. Furthermore, utilization of incident CAC, as opposed to hard clinical events, would allow for a markedly less costly trial design assessing the efficacy of such a pharmacogenomic algorithm.

## Abbreviations

(AU): Agatston units; (CAC): coronary artery calcium; (CAD): coronary artery disease; (Hp): Haptoglobin; (Hu): Hounsfield units.

## Competing interests

The authors declare that they have no competing interests.

## Authors' contributions

MS analyzed the data that are reported here and wrote this manuscript. JSB and GK collected patient data, assisted in the analysis and reporting of the data herein, and made editorial contributions to the manuscript. MR designed and supervised the CACTI study, and made extensive scientific and editorial contributions to this manuscript. OL, RML, and YA genotyped participant samples for Haptoglobin and made editorial contributions to the manuscript. AL developed the method for Haptoglobin genotyping, assisted in data analysis, and made extensive scientific and editorial contributions to this manuscript. All authors have read and approved the final manuscript.
